# Meaningful Use of Pathogen Genomic Data

**DOI:** 10.1128/mbio.00311-22

**Published:** 2022-04-25

**Authors:** Taj Azarian

**Affiliations:** a Burnett School of Biomedical Sciences, University of Central Florida, Orlando, Florida, USA

**Keywords:** genomic databases, genomic epidemiology, MRSA, population genomics, Staphylococcus aureus, bioinformatics

## Abstract

Population genomic analysis is a powerful tool to understand the evolutionary history of pathogens and the factors contributing to the success or failure of lineages. These studies have significant implications for human health, as evident from our ongoing tracking of SARS-CoV-2. In their article, Gill et al. (J. L. Gill, J. Hedge, D. J. Wilson, and R. C. MacLean, mBio 12:e02168-21, 2021, https://doi.org/10.1128/mBio.02168-21) demonstrate the utility of pathogen genomic data by comprehensively elucidating the origin of methicillin-resistant Staphylococcus aureus ST239. To accomplish this, they leveraged newly developed tools for querying large genomic data sets. Overall, these analyses rely on the availability of representative genomic data along with their associated metadata—information about where and when samples were collected, clinical and epidemiological characteristics, and phenotypic properties. However, in many instances, these data are missing. Here, I borrow the term “meaningful use” from the Health IT field to describe the need to maximize the utility of genomic data and make suggestions for how to address the current limitations.

## COMMENTARY

A common goal among population genomic studies is to understand the evolutionary history of a pathogen and the relative success of its lineages. These approaches are dependent on access to pathogen genomic data, rich metadata, and preferably the isolate and/or relevant phenotypic profiling. In their recent mBio publication, Gill et al. provide a clear example of how, when available, such data can be applied to answering unresolved questions, thereby increasing the utility of genomic data beyond their initial purpose (1). The authors analyzed published Staphylococcus aureus genomic data spanning 70 years to understand the origins of ST239, a well known methicillin-resistant S. aureus (MRSA) strain. After its emergence, MRSA ST239 became a leading cause of health care-associated infections, highly prevalent and globally distributed, except in the United States, where it has remained relatively rare. Before their work, it was proposed that ST239 arose from a large recombination event between ST8 and ST30 strains (2). By querying representative genomes of ST30 and ST8, the authors were able to provide more definitive evidence of this hybrid evolution. They showed that the genomic backbone was most related to ST8, while the acquired region originated from an ST30 lineage that evolved from the phage type 80/81 clone, a notorious strain of methicillin-susceptible S. aureus (MSSA) that frequently caused hospital outbreaks in the 1950s and 1960s. As the SCC*mec* type III element, which confers resistance to multiple antibiotics, was not found among ∼1,900 published ST30 genomes, they posit that it was acquired from an ST30 ancestor after divergence of the ST239 lineage. Furthermore, they were able to date the origin of ST239 MRSA to between 1920 and 1945, echoing findings from another group that recently showed that methicillin-resistant S. aureus emerged prior to the clinical introduction of methicillin (3). To explain the recent decrease in prevalence of ST239, the authors investigated the competitive fitness of ST239, finding it lower than its ST30 and ST8 progenitors. To reinforce this finding, they assessed selective forces acting on the genomic backbone and acquired regions, finding a fitness cost associated with acquisition of genes coding for antimicrobial resistance. Nevertheless, it remains unclear whether the decline of ST239 was the result of direct competition with other lineages or some combination of competition with improvements in infection prevention in the health care setting and decreased selective pressure as the result of antibiotic stewardship.

Although the limited geographic distribution of ST239 was not specifically addressed by their analysis, Gill and colleagues also provide a putative explanation for why it was never a successful strain in North America. In the United States, USA100 and USA800 belonging to ST5 and USA500 belonging to ST8 were historically the leading causes of health care-associated MRSA infections (4). These lineages emerged in the Western Hemisphere at approximately the same time that ST239 was emerging elsewhere. The emergence of the highly successful ST8 USA300 North American epidemic clone in the 1990s likely ensured that ST239 would never become established (5). This was supported by the authors’ findings that ST8 demonstrated greater fitness and the observation that the few sequenced ST239 North American isolates were interspersed throughout the ST239 global phylogeny, suggesting multiple introductions that never took hold. Taken together, their findings provide insight into the fate of prevalent lineages and considerably advance our understanding about how new lineages emerge, how selection may act on different parts of the genome, and why we observe considerable geographic variation in lineage distribution. More work is needed to investigate the relative contribution of human interventions and strain competition, especially among MRSA and MSSA lineages. Overall, the authors present an eloquent synthesis of computational and experimental approaches combined with genomic detective work, highlighting the promise of pathogen genomic data meta-analysis.

**FIG 1:**
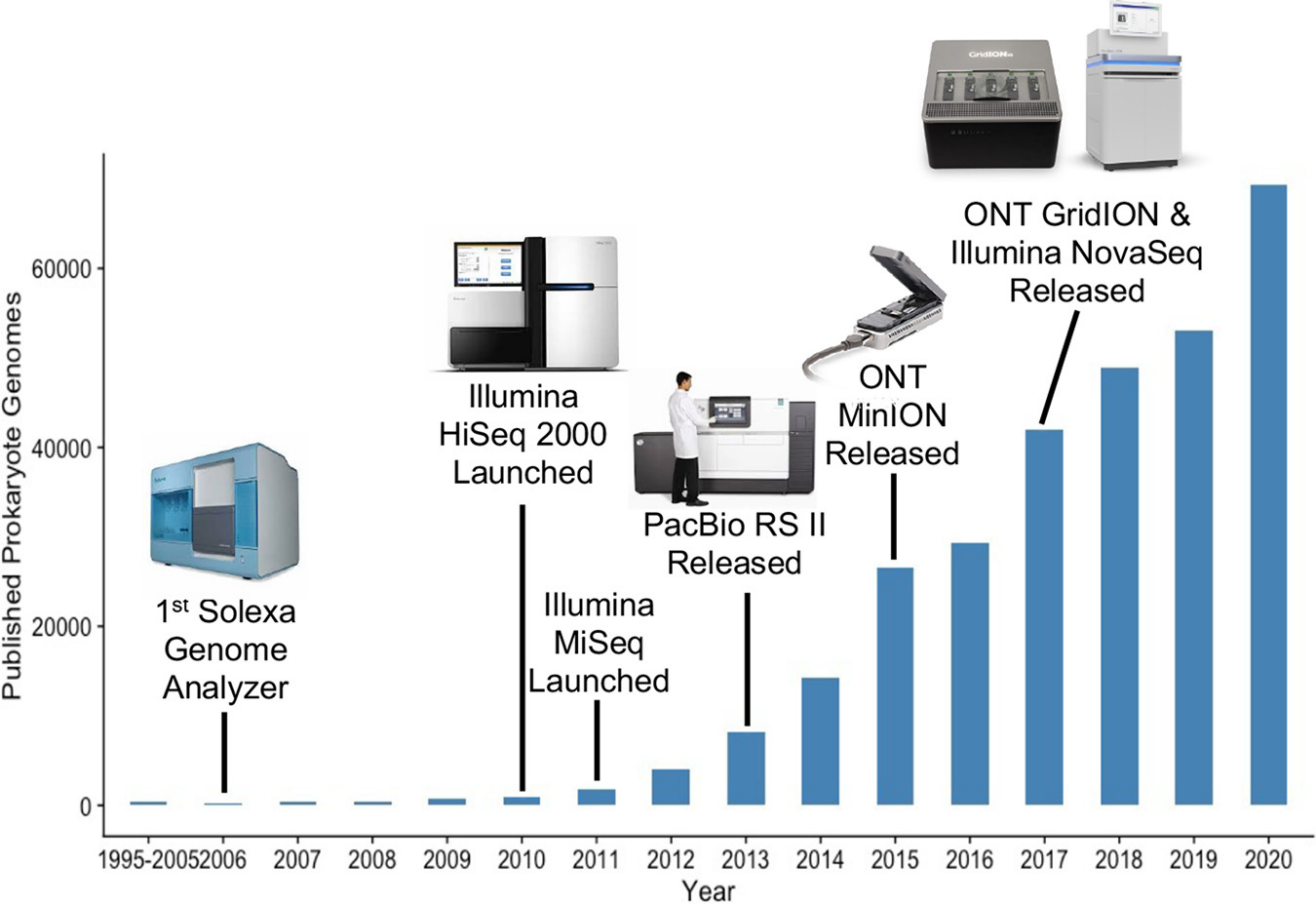
Yearly published draft prokaryotic genomes published in the National Center for Biotechnology Information (NCBI) database (https://ftp.ncbi.nlm.nih.gov/genomes/GENOME_REPORTS/prokaryotes.txt). The release dates of notable sequencing platforms are noted on the figure. ONT, Oxford Nanopore Technologies.

The present analysis was made possible by the wealth of published sequencing data on S. aureus, which is one of the most well represented bacterial species in publicly available genomic data repositories (6). Over the last decade, the advances in sequencing technology and accompanying reduction in sequencing costs have resulted in an exponential increase in the number of published microbial data sets (Fig. 1) (7). However, the vast majority of these data exist as raw sequencing experiments deposited in the European Nucleotide Archive (ENA) and/or NCBI Sequence Read Archive (SRA), which makes them less accessible for routine querying. This limitation, compounded by the scarcity of available metadata, has diminished the utility of genomic data for secondary studies. In particular, it has been difficult for researchers to identify relevant data sets (e.g., genomes of samples collected from a disease type/sampling site, date range, or location) or search genomes for features of interest such as a specific genotype, virulence or antibiotic resistance determinants, or other mobile genetic element. Often this difficulty results in the generation of new genomic data when existing data may have sufficed had metadata been made available. The ideal genomic database would offer more comprehensive and easily searchable metadata, which would enable improved tracking of the emergence and spread of epidemiologically important pathogens, elucidate the factors contributing to emergence, and facilitate the identification of novel mechanisms for virulence and antimicrobial resistance.

Current limitations are partially being addressed by significant methodological advances in development of computational tools for handling the ever-growing amount of data. Two such tools, BIGSI (https://bigsi.readme.io/) and Staphopia (https://staphopia.emory.edu/), were used in the present study (8, 9). BIGSI allows for efficient indexing and rapid querying of large genomic databases. As an early proof of concept, the authors indexed the raw sequencing data in the entire ENA database, while in subsequent iterations these data were assembled into draft genomes and then indexed (6). As a result, researchers were no longer limited to searching the ∼330,000 published draft prokaryotic genomes in NCBI/ENA but also the >660,000 genomes that previously existed only as raw sequencing data (6). Staphopia houses a centralized database for S. aureus genomes and pathogen-specific results such as genotype and antibiotic resistance and virulence gene profiles. Its successor Bactopia (https://bactopia.github.io/) provides an analysis framework that can be easily applied to other pathogens. In combination, these tools allowed the authors of the present study to easily identify all published genomes belonging to ST239, ST30, and ST8 in the Staphopia database and then use BIGSI to search all microbial genomic data for the SCC*mec*-III mobile genomic element sequence found in ST239. Simply put, their analysis would not have been possible or as comprehensive without these tools.

During the development of Staphopia, the authors also highlighted two common limitations of large genomic databases: accessible metadata and geographic representation. In their work, Petit and Read found that of the ∼43,000 S. aureus samples deposited between 2010 and 2017, only 40% had a collection date, 35% had a geographic location, and 35% had an isolate source (9). Only 28% of samples could be linked to a publication, and while most journals require genomic data to be deposited and the metadata published, there are minimal standards for which fields are required. Even if rich metadata were included with associated publications, it is tedious for researchers to identify relevant genomic studies and then aggregate largely incomplete and inconsistent metadata. A recent secondary analysis of S. aureus genomic data we performed utilized 436 genomes spanning 45 published studies and 55 NCBI bioprojects (10). This required resource intensive abstraction of metadata, and in many instances, the study authors were contacted to obtain the requisite data for meaningful analysis. Finally, by assessing the available metadata, it is evident that there is a strong geographic bias among published genomic data, with much of it comprised of samples collected from North America and Western Europe. This has clear implications for population genomic studies. For example, accurately tracking the demographic history of a pathogen or identifying the origin of a recombination block relies on having representative sampling of the ancestor within the data set.

Myriad entities such as private laboratories, public health agencies, and academic researchers routinely generate sequencing data, and reasons can vary for not publishing detailed metadata, including (i) privacy concerns, (ii) the desire to use the data to its fullest extent before others gain access, (iii) wanting credit for invested resources, and (iv) a feeling of inequity among data generators. Furthermore, when data are generated by a public health entity, common data elements such as date of collection or location can be considered protected health information released at the discretion of the agency. The debate about genomic data-sharing practices and policies has been brought to the forefront of scientific discussion during the SARS-CoV-2 pandemic (11). There are strong arguments from groups advocating for unrestricted data access and others wanting a more conservative approach. Moving forward, the scientific community should revisit how sequencing data are published along with their associated metadata. Likely, a top-down and bottom-up approach will be needed to address the current limitations. While the National Institutes of Health (NIH) published a genomic data-sharing policy in 2015 that set requirements for the publication of data generated through federal funding, the metadata requirements are relatively minimal (12). Further, the disparity between the number of published assembled draft genomes and those stored as raw sequencing data is partially the result of the current policy, which requires that genomic data be deposited within 45 days after generation (13). This approach favors rapid, prepublication data availability, which undeniably is necessary for responding to public health emergencies. However, the proper collection and curation of metadata requires considerable effort and is frequently performed during preparation for publication. The unintended result is a less than optimal genomic database. Currently, NIH is revisiting this policy and has posted a request for information, providing an opportunity for stakeholders in the fields of genomics, infectious diseases, public health, ecology, microbiology, and epidemiology to shape the long-term vision and balance acceptability with usability.

As a community of scholars with a vested interest in access to useful microbial genomic data, we can improve our practices by publishing detailed metadata with their associated sequence data. While a minimal acceptable data set is not well established, at the least, the year and month of collection, location of collection (at a minimum region/state/province and country), source (e.g., human, fomite, or animal; carriage or disease if from a host), and disease type should be included. Ideally, phenotypic properties that cannot be inferred from the genomic data such as antibiotic susceptibility should be included. Further, as more complete metadata and draft genome assemblies are generated during analysis, these data should be appended to the primary submission, and as a community, we should determine whether policies requiring this are needed. Most importantly, metadata should not be relegated to an unsearchable table in a supplemental pdf file. If more detailed metadata are available that are not compatible with current data structures, repositories like Data Dryad (https://datadryad.org/) may provide an alternative to supplemental metadata tables. Retrospectively, we can leverage computational advances to scrape metadata from publications and link them to accession numbers of genomic data sets. This may be most useful for pathogen-specific databases such as Staphopia. Finally, in terms of increasing the geographic representativeness of genomic data, efforts like that of the Global Pneumococcal Sequencing Project are a model for engaging countries with limited resources and ensuring that their participation is equitable (14). Together, these efforts would greatly improve the richness and utility of genomic data sets and make analysis such as the one by Gill et al. (1) more commonplace.

## References

[1] Gill JL, Hedge J, Wilson DJ, MacLean RC. 2021. Evolutionary processes driving the rise and fall of *Staphylococcus aureus* ST239, a dominant hybrid pathogen. mBio 12:e02168-21. 10.1128/mBio.02168-21 PMC8669471 34903061

[2] Robinson DA, Enright MC. 2004. Evolution of *Staphylococcus aureus* by large chromosomal replacements. J Bacteriol 186:1060–1064. doi:10.1128/JB.186.4.1060-1064.2004. 14762000 PMC344219

[3] Larsen J, Raisen CL, Ba X, Sadgrove NJ, Padilla-González GF, Simmonds MSJ, Loncaric I, Kerschner H, Apfalter P, Hartl R, Deplano A, Vandendriessche S, Bolfíková BC, Hulva P, Arendrup MC, Hare RK, Barnadas C, Stegger M, Sieber RN, Skov RL, Petersen A, Angen Ø, Rasmussen SL, Espinosa-Gongora C, Aarestrup FM, Lindholm LJ, Nykäsenoja SM, Laurent F, Becker K, Walther B, Kehrenberg C, Cuny C, Layer F, Werner G, Witte W, Stamm I, Moroni P, Jørgensen HJ, de Lencastrea H, Cercenado E, García-Garrote F, Börjesson S, Hæggman S, Perreten V, Teale CJ, Waller AS, Pichon B, Curran MD, Ellington MJ, Welch JJ, et al. 2022. Emergence of methicillin resistance predates the clinical use of antibiotics. Nature 602:135–141. doi:10.1038/s41586-021-04265-w. 34987223 PMC8810379

[4] McDougal LK, Steward CD, Killgore GE, Chaitram JM, McAllister SK, Tenover FC. 2003. Pulsed-field gel electrophoresis typing of oxacillin-resistant *Staphylococcus aureus* isolates from the United States: establishing a national database. J Clin Microbiol 41:5113–5120. doi:10.1128/JCM.41.11.5113-5120.2003. 14605147 PMC262524

[5] Challagundla L, Luo X, Tickler IA, Didelot X, Coleman DC, Shore AC, Coombs GW, Sordelli DO, Brown EL, Skov R, Larsen AR, Reyes J, Robledo IE, Vazquez GJ, Rivera R, Fey PD, Stevenson K, Wang S-H, Kreiswirth BN, Mediavilla JR, Arias CA, Planet PJ, Nolan RL, Tenover FC, Goering RV, Robinson DA. 2018. Range expansion and the origin of USA300 North American epidemic methicillin-resistant *Staphylococcus aureus*. mBio 9:e02016-17. doi:10.1128/mBio.02016-17. PMC5750399 29295910

[6] Blackwell GA, Hunt M, Malone KM, Lima L, Horesh G, Alako BTF, Thomson NR, Iqbal Z. 2021. Exploring bacterial diversity via a curated and searchable snapshot of archived DNA sequences. PLoS Biol 19:e3001421. doi:10.1371/journal.pbio.3001421. 34752446 PMC8577725

[7] National Human Genome Research Institute. 2020. DNA sequencing costs: data. National Human Genome Research Institute website. https://www.genome.gov/about-genomics/fact-sheets/DNA-Sequencing-Costs-Data.

[8] Bradley P, den Bakker HC, Rocha EPC, McVean G, Iqbal Z. 2019. Ultrafast search of all deposited bacterial and viral genomic data. Nat Biotechnol 37:152–159. doi:10.1038/s41587-018-0010-1. 30718882 PMC6420049

[9] Petit RA, Read TD. 2018. *Staphylococcus aureus* viewed from the perspective of 40,000+ genomes. PeerJ 6:e5261. doi:10.7717/peerj.5261. 30013858 PMC6046195

[10] Azarian T, Cella E, Baines SL, Shumaker MJ, Samel C, Jubair M, Pegues DA, David MZ. 2021. Genomic epidemiology and global population structure of exfoliative toxin A-producing *Staphylococcus aureus* strains associated with staphylococcal scalded skin syndrome. Front Microbiol 12:663831. doi:10.3389/fmicb.2021.663831. 34489877 PMC8416508

[11] Maxmen A. 2021. Why some researchers oppose unrestricted sharing of coronavirus genome data. Nature 593:176–177. doi:10.1038/d41586-021-01194-6. 33953391

[12] National Institutes of Health Office of Science Policy. NIH Genomic Data Sharing. National Institutes of Health website. https://osp.od.nih.gov/scientific-sharing/genomic-data-sharing/.

[13] National Institute of Allergy and Infectious Diseases, National Institutes of Health. Data Management and Sharing Guidelines. National Institutes of Health website. https://www.niaid.nih.gov/research/data-sharing-guidelines.

[14] Bentley SD, Lo SW. 2021. Global genomic pathogen surveillance to inform vaccine strategies: a decade-long expedition in pneumococcal genomics. Genome Med 13:84. doi:10.1186/s13073-021-00901-2. 34001237 PMC8130287

